# Gene therapy for pediatric genetic kidney diseases

**DOI:** 10.1002/pdi3.16

**Published:** 2023-06-10

**Authors:** Yi Lu, Yandong Song, Shaokai Sun, Lirong Zhang, Yupeng Chen

**Affiliations:** ^1^ Key Laboratory of Immune Microenvironment and Disease (Ministry of Education) The Province and Ministry Co‐sponsored Collaborative Innovation Center for Medical Epigenetics Department of Biochemistry and Molecular Biology School of Basic Medical Sciences Tianjin Institute of Urology The Second Hospital of Tianjin Medical University Tianjin Medical University Tianjin China; ^2^ School of Medical Imaging Tianjin Medical University Tianjin China

**Keywords:** AAV, Alport syndrome, CRISPR‐Cas9, gene therapy, LNP, pediatric genetic kidney diseases, PKD

## Abstract

Genetic kidney disease is the main cause of chronic kidney disease in children. While the pathogenic genes associated with most genetic kidney diseases have been identified, the underlying mechanisms of disease initiation remain ambiguous, and effective treatment modalities are limited. Gene therapy has emerged as a promising approach for treating genetic diseases. Several gene therapy drugs have been successfully launched to treat genetic diseases affecting the eyes and muscles. However, the development of gene therapy agents for kidney diseases lags behind that of other genetic disorders. In this review, we first comprehensively summarize the main gene therapy strategies. Next, we provide an overview of current treatment options as well as the latest research on gene therapy approaches for pediatric genetic kidney diseases with a particular emphasis on Alport syndrome and polycystic kidney disease. Finally, we discuss the challenges and potential solutions for gene therapy of pediatric genetic kidney diseases.

## Graphical abstract

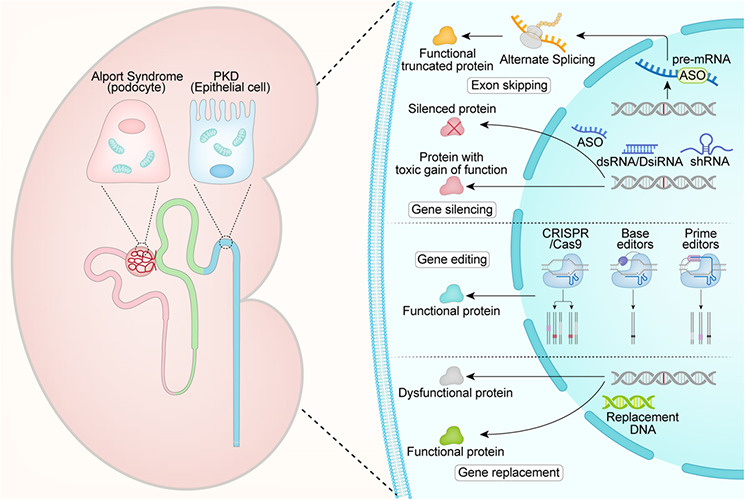

## 1 INTRODUCTION

Chronic kidney disease (CKD) refers to a pathological condition characterized by anomalous renal structure and function, which is correlated with reduced quality of life and increased mortality.^1^ Genetic factors contribute greatly to CKD pathogenesis, particularly in the pediatric population, where genetic kidney diseases account for approximately 30% of cases.^2^ Genetic kidney disease in children often progresses to end‐stage kidney disease (ESKD) due to its non‐specific clinical manifestations, low detection rates, involvement of multiple pathogenic genes, and poor response to existing treatment drugs. Thus, there is a pressing need to develop drugs tailored to address pediatric genetic kidney diseases.

Gene therapy is a promising approach for treating genetic diseases. Gene therapy strategies include nucleic acid‐mediated gene silencing and exon skipping, nuclease‐mediated gene knockout and repair, and functional gene delivery. Currently, the most frequently used gene therapy strategies in preclinical research for genetic diseases are gene editing using CRISPR/Cas9 and its derivatives as tools, as well as functional gene delivery.^3^, ^4^ In animal models, viral and non‐viral vectors have been successfully used to deliver gene therapy drugs for targeted repair of multiple organs, including the eyes, liver, brain, and skeletal muscles.^5^, ^6^, ^7^

Renal compensatory mechanisms are known to be robust, such that restoration of a portion of kidney units can sustain renal function, rendering genetic kidney disease a suitable indication for gene therapy. However, despite substantial advances in the field, there have been no reported successful cases of gene therapy for the kidneys. Pediatric genetic kidney diseases comprise a broad range of disorders, including genetic glomerular diseases, genetic tubular diseases, ciliopathies, congenital urinary tract abnormalities, and genetic metabolic diseases.^8^

In this review, we focus on genetic glomerular diseases and genetic tubular diseases, we first present the current gene therapy strategies employed for genetic diseases. Then, we summarize the clinical treatment options and the gene therapy drugs currently under development for genetic kidney diseases. Lastly, we propose future directions and discuss the challenges faced in gene therapy drugs for pediatric genetic kidney diseases.

## 2 GENE THERAPY STRATEGIES FOR GENETIC DISEASES

### 2.1 Delivering gene editing tools to correct pathogenic mutations

Gene editing is a molecular technology that involves deleting, inserting, or replacing a specific segment or specific nucleotides of the genome to induce specific changes in the genome. Over the last 2 decades, the field of gene editing has witnessed remarkable advancements with the emergence of various technologies, such as meganucleases, zinc finger nucleases, transcription activator‐like effector nucleases, and the CRISPR‐associated (Cas) nuclease system.^9^ Among them, CRISPR/Cas9 has become the top choice for gene editing due to its advantages of low cost, easy manipulation, and high efficiency.

CRISPR/Cas9 is an RNA‐guided DNA nuclease system that has evolved as an adaptive immune mechanism in prokaryotes.^9^ Typically, the CRISPR/Cas9 system is composed of a single‐stranded RNA (sgRNA) and Cas9 protein. The sgRNA directs the nuclease to a specific DNA site, making the system modular and easy to engineer. Upon binding to the genomic site of interest, the CRISPR/Cas9 system generates double‐strand breaks (DSBs). These breaks can be repaired through two general pathways: Non‐homologous end joining (NHEJ) and homology directed repair (HDR). NHEJ is the predominant random repair mechanism, resulting in in‐frame amino acid deletions, insertions, or frameshift mutations. In contrast, HDR can generate precise repair at the target sites in the presence of exogenous templates, making it more suitable for gene repair than NHEJ despite having a lower efficiency.^10^, ^11^ CRISPR/Cas9 technology has exhibited substantial potential in the therapeutic management of genetic disorders. Several proof‐of‐concept investigations utilizing animal models of Duchenne muscular dystrophy (DMD), caused by mutations in the *DMD* gene, have successfully demonstrated the restoration of functional dystrophin via gene editing.^12^ These data suggest that CRISPR/Cas9‐based gene editing holds promise for the treatment of human genetic diseases.^13^

**FIGURE 1:**
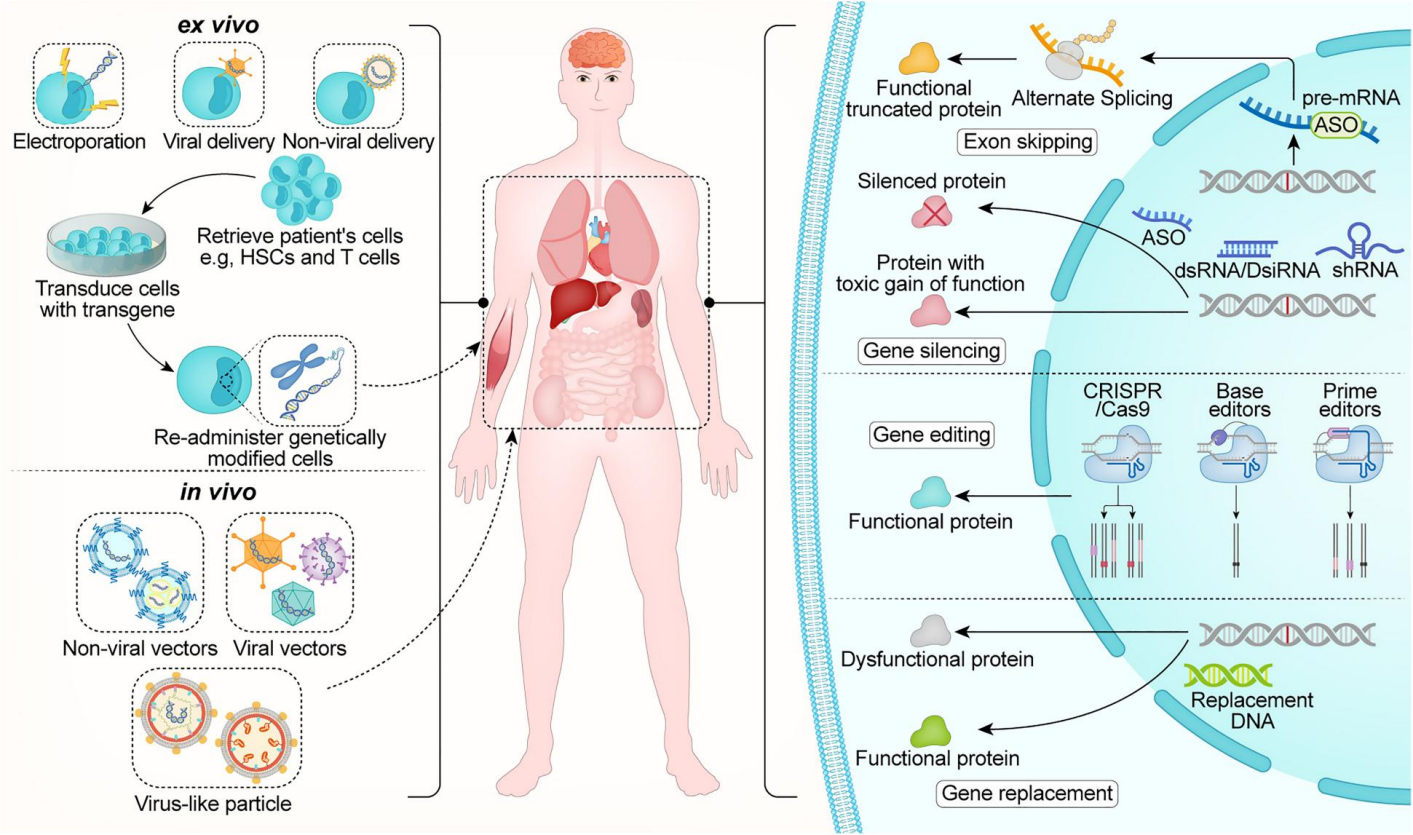
The current main strategies for gene therapy. Currently, the main delivery methods for ex vivo gene therapy are electroporation, virus‐based vectors, and non‐viral vectors. Cells that have undergone gene editing or modification are then reinfused into the patient's body, generally for the treatment of hematologic diseases. The main delivery methods for in vivo gene therapy are nanoparticle‐based non‐viral vectors, virus‐based vectors (adenovirus, lentivirus, AAV), and viral‐like particles. The main cargo for gene therapy includes functional genes, CRISPR/Cas9‐derived gene editing tools, and small nucleic acid drugs (ASO, RNAi). ASO, antisense oligonucleotides; RNAi, RNA interference.

Currently, point mutations are recognized as the most prominent known source of human pathogenic mutations.^14^ Base editing (BE) is a technique developed to efficiently and precisely correct single nucleotide mutations. This technology is capable of producing precise point mutations without the need for DNA donor templates or reliance on cellular HDR, all while avoiding the creation of DSBs. DNA base editors can be broadly classified into two groups: adenine base editors (ABEs), which convert an A‐T base pair to a G‐C base pair,^14^, ^15^ and cytosine base editors (CBEs), which convert a C‐G base pair to a T‐A base pair.^16^, ^17^ Recent research has resulted in the development of new base editors capable of performing C•G to G•C (CGBEs) transversions.^18^, ^19^ BEs have been used therapeutically to correct disease‐causing point mutations that rescue disease phenotypes (Figure 1). Yeh et al. have developed a CBE strategy to treat Baringo mice, which carry a homozygous missense point mutation (A545G) in *Tmc1* resulting in deafness.^20^ In vivo repair of *Tmc1* restored the sensory transduction in inner hair cells and hair cell morphology.^20^ Suh et al. have developed an ABE strategy to treat rd12 mice (C130T) carrying a nonsense mutation in *RPE65* resulting in visual impairment.^21^ ABE‐mediated repair of *RPE65* partially restored the visual function in the mice.^21^

While BEs can effectively correct a large proportion of pathogenic single‐nucleotide polymorphisms, they have limitations in their ability to perform all possible single‐nucleotide conversions or repair fragment insertions or deletions. To overcome these limitations, Anzalone et al. developed prime editors (PEs), which enable random single‐nucleotide transitions, small insertions, and small deletions without generating DSBs.^22^ PEs consist of a fusion of reverse transcriptase and Cas9 nickase domain and utilize an engineered prime editing guide RNA (pegRNA). PegRNA directs the Cas9 nickase to a specific target locus and encodes a specific editing sequence of interest. Multiple studies have reported the use of PEs for in vivo gene editing to treat diseases^23^, ^24^, ^25^ (Figure 1). For example, Liu et al. injected PE2 to correct the pathogenic mutation in the Alpha‐1 antitrypsin deficiency (AATD) mouse model.^26^ Jang et al. used PE2 and PE3 to treat hereditary tyrosinemia and Leber congenital amaurosis in mice, precisely correcting the pathogenic mutations and improving the disease phenotype without detecting off‐target editing.^23^

### 2.2 Delivering functional genes to compensate the non‐functional mutated genes

Genetic diseases are caused by gene mutations, and for most of these diseases, the pathogenic genes are well understood. Besides correcting mutations at specific sites, the delivery of functional genes capable of producing functional proteins can also correct gene abnormalities (as shown in Figure 1). The delivery of functional genes is generally considered safer than gene editing, which may cause potential off‐target effects and lead to adverse events in vivo. Functional gene delivery has been successfully applied in several clinical studies for genetic diseases, such as recessive dystrophic epidermolysis bullosa (RDEB), a rare condition caused by mutations in the *COL7A1* gene. Such mutations cause skin fragility and blistering, leading to wound formation and scarring.^27^, ^28^ Beremagene geperpavec (B‐VEC), a local application of a herpes simplex virus type 1 (HSV‐1)‐based gene therapy, is designed to restore C7 protein by delivering a normal copy of *COL7A1* gene.^29^ Clinical studies have confirmed that RDEB patients receiving B‐VEC treatment experienced wound healing at 3 and 6 months with only mild systemic side effects and itching.^30^

AAV is currently the preferred vector for delivering functional genes in the treatment of genetic diseases due to its long‐term expression, low integration ability, and low immunogenicity.^31^ Luxturna, a gene therapy drug developed by Spark Therapeutics, is an AAV‐based gene therapy that delivers a normal copy of the *RPE65* gene directly to retinal cells. These retinal cells then produce normal RPE65 proteins, which convert light into electrical signals in the retina, restoring vision in patients with genetic retinal dystrophy.^32^, ^33^ In 2017, Luxturna became the first gene therapy drug to receive FDA approval, signaling an important milestone in the field of gene therapy. This approval highlights the potential for gene therapy to play a crucial role in treating numerous genetic diseases.

### 2.3 Delivering therapeutic nucleic acids to modulate pathogenic gene expression

Nucleic acid therapy aims to achieve therapeutic effects through gene inhibition, addition, replacement, and editing. The most commonly used methods of nucleic acid therapy include antisense oligonucleotides (ASO) and RNA interference (RNAi) (as shown in Figure 1).^34^, ^35^

ASO is a short synthetic nucleic acid that can regulate protein synthesis after transcription through various mechanisms, such as modifying pre‐mRNA processing and splicing, competitive inhibition, spatial blocking of translation mechanisms, and binding target RNA degradation. Chemical modifications of ASO nucleotides, bases, and backbone are essential to improve pharmacokinetics and pharmacodynamics while maintaining target affinity and efficacy.^36^, ^37^ Currently, a series of ASO drugs have been developed for DMD and spinal muscular atrophy (SMA),^38^, ^39^ most of which are based on inducing exon skipping and intron inclusion by binding to pre‐mRNA.

RNAi is a biological process that regulates gene expression by silencing or suppressing the activity of specific genes. It is mediated by small RNA molecules, including small interfering RNAs (siRNAs) and microRNAs (miRNAs). siRNAs are short double‐stranded RNA molecules composed of approximately 20 nucleotides, which can silence the expression of target genes. When siRNAs enter a cell, they are processed into sgRNA that bind to the RNA‐induced silencing complex (RISC). The RISC complex guides the siRNAs to complementary mRNAs and cleaves them into small fragments, leading to the suppression of gene expression.^40^ Currently, a number of siRNA drugs have been approved for clinical use. For instance, Patisiran was the first siRNA drug to receive approval for market release. It targets transthyretin (TTR) and is used to treat hereditary ATTR amyloidosis.^41^ Givosiran is another siRNA drug that targets δ‐aminolevulinate synthase 1 (ALAS1) and is used to treat acute intermittent porphyria.^42^ Inclisiran, on the other hand, targets the PCSK9 gene and is used to treat hypercholesterolemia. Lumasiran is an siRNA drug that targets the glycolate oxidase (GO) gene and is used to treat primary hyperoxaluria type 1.^42^

miRNAs are small single‐strand RNA molecules that are approximately 20–22 nucleotides in length. miRNAs are synthesized by endogenous genes within cells. After miRNAs bind to RISC, they can guide RISC to bind to mRNAs, thereby affecting the stability and translation of mRNAs and ultimately influencing the expression of target genes.^40^ miRNA mimic enhances the function of endogenous miRNA, while antimiR inhibits the function of miRNAs. Nonetheless, developing miRNA drugs faces challenges, such as increasing their stability and optimizing the drug delivery system.^43^, ^44^, ^45^

### 2.4 Delivery vectors for gene therapy

Gene therapy vectors are mainly divided into non‐viral vectors, viral vectors, and viral‐like particles (Figure 1). Lipid nanoparticles (LNPs) are currently the most widely used non‐viral vectors, which can effectively deliver ribonucleoproteins (RNPs), siRNAs, and mRNAs.^46^, ^47^ LNPs are fully synthetic vectors that are typically composed of four components: cationic or ionizable lipids, helper lipids, polyethylene glycol (PEG) lipids, and cholesterol. The synthesis process for LNPs is mature and straightforward. These vectors enter cells through endocytosis and then escape from endosomes by disrupting the endosomal membranes, ultimately entering the cytosol of the target cells. In recent years, multiple studies have found that changing the components of LNPs can achieve targeted delivery to different cells or organs.^48^, ^49^ LNPs offer several advantages over viral vectors, especially in the delivery of gene editors. LNPs delivery leads to the transient expression of gene editors, which reduces the likelihood of off‐target editing compared to the prolonged expression of viral genomes.^50^, ^51^ Furthermore, because LNPs are synthetic carriers, they have lower immunogenicity than viral vectors. Many benefits of LNPs have resulted in encouraging results in the development of LNP‐based gene therapies for a variety of genetic diseases, making them an increasingly popular choice for many clinical gene therapies. Looking ahead, the development of LNPs capable of efficient non‐liver delivery remains a key goal in the field of therapeutic gene editing.

Viral delivery vectors can be categorized into three primary types: lentiviral vectors, adenoviral vectors, and AAV vectors. Viruses have evolved to overcome the barriers of intracellular delivery and can naturally transport nucleic acid cargoes to many cell types. Due to these advantageous characteristics, viruses are promising vehicles for gene editors, and many viral vectors have been developed for in vivo gene therapy applications. A majority of in vivo gene editing applications use AAV as the gene therapy vector, while some preclinical studies use lentivirus or adenoviruses.^31^ AAV is a 25‐nm non‐enveloped virus composed of 60 copies of viral proteins VP1, VP2, and VP3 assembled into an icosahedral capsid. Its packaging capacity is limited to 4.9 kb.^52^, ^53^ AAV is generally considered to be safe and biocompatible. AAV can also target multiple organs, including the eyes, liver, brain, heart, and skeletal muscle.^54^, ^55^ Additionally, new AAV subtypes with different targeting capabilities can be obtained through laboratory evolution and rational engineering.^56^, ^57^ However, the limited packaging capacity of AAV currently presents the biggest challenge, leading to the ongoing development of gene editors and functional genes that meet AAV's packaging requirements. The use of lentivirus as vectors for in vivo gene therapy has some important drawbacks, including concerns about genomic integration, genetic toxicity, immunogenicity, and high manufacturing costs, all of which may limit their use in clinical applications.^58^, ^59^ Adenoviruses are currently the most widely used viral vectors in global gene therapy clinical trials (>20%), mainly due to their large packaging capacity, good genetic stability, high transduction efficiency, and the ability to rapidly produce large amounts of virus.^60^ However, the high immunogenicity of adenoviruses cannot be ignored.

Virus‐like particles (VLPs) have emerged as a promising delivery vehicle for gene editors. VLPs are non‐infectious particles assembled from viral proteins that can package the desired payload of mRNA, proteins, or RNPs, either instead of or in addition to viral genetic materials.^61^ Because VLPs are derived from existing viral scaffolds, they leverage the natural properties of viruses for efficient intracellular delivery, including their ability to package payloads, escape endosomes, and be reprogrammed to target different cell types. However, unlike viruses, VLPs deliver gene editors as mRNA or protein rather than DNA, greatly reducing the risk of off‐target gene editing and viral genome integration.^61^ Therefore, VLPs are an attractive vehicle for delivery of gene editing agents as they can provide the key advantages of both viral and non‐viral delivery. Several studies have shown that the cell‐type specificity of VLPs can be altered in vitro by using different envelope glycoproteins, leading to the development of organ‐specific VLP vectors.^62^, ^63^ While several research groups have shown that systemic administration of VLPs is non‐toxic to mice, further research is needed to investigate the safety and feasibility of VLPs in various disease models, including large animal models in the future.

### 2.5 Gene therapy operation

There are two modes of operation for gene therapy: ex vivo gene therapy and in vivo gene therapy. Ex vivo gene therapy is a gene therapy technique in which a patient's cells are removed and genetically modified outside the body, and then the modified cells are reintroduced into the patient's body to achieve the goal of treating the disease.^64^ This treatment is typically used to treat certain hematologic diseases, such as severe combined immunodeficiency or sickle cell anemia. In this treatment, the patient's own hematopoietic stem cells or immune cells are collected and genetically modified in vitro to produce normal proteins or immune cells and then reintroduced into the patient's body to restore the immune function or alleviate disease symptoms. Compared to other gene therapy methods, ex vivo gene therapy is safer because the gene modification process takes place outside the body, avoiding the effects on other cells in the patient's body, and the safety of the modified cells can be ensured by testing them before reintroduction.

In vivo gene therapy approach involves the administration of gene therapy vectors, such as viruses or nanoparticles, directly into the patient's body for the purpose of delivering therapeutic genes to repair or replace missing or abnormal genes.^64^ Typically, this method is utilized for the treatment of certain genetic disorders or cancers. Upon injection, the gene therapy vectors transfer the therapeutic genes to the patient's cells. These therapeutic genes then aid in repairing or replacing missing or abnormal genes, thereby achieving the intended goal of treating the disease. In contrast to ex vivo gene therapy, in vivo gene therapy offers greater convenience since it does not require the collection and processing of the patient's cells and can be performed directly within the patient's body. However, this approach also carries potential risks, including the possibility of immune responses or adverse reactions to the gene therapy vectors. As a result, rigorous evaluations of safety and efficacy are essential prior to the administration of in vivo gene therapy.

## 3 CURRENT THERAPEUTIC STATUS FOR PEDIATRIC GENETIC KIDNEY DISEASES

Genetic diseases caused by single‐gene mutations are relatively easier to treat with gene therapy as compared to those caused by multiple gene mutations. This is because single‐gene mutations involve a specific genetic alteration in one gene, making it easier to identify and target the affected gene for treatment. In the context of genetic kidney diseases in children, two common examples are Alport syndrome and PKD.

### 3.1 Current therapies for Alport syndrome

Alport syndrome is a genetic kidney disease that affects the glomerular basement membrane (GBM), which is responsible for filtering blood in the kidneys. It is one of the most common genetic kidney diseases and is clinically characterized by hematuria and progressive decline in kidney function.^65^ In addition, some patients may also experience sensorineural hearing loss and abnormal changes in the eyes.^65^, ^66^

Alport syndrome is caused by mutations in the genes that encode for type IV collagen alpha‐chains of the GBM. The inheritance patterns include X‐linked dominant, autosomal recessive, and autosomal dominant. X‐linked dominant inheritance caused by mutations in the *COL4A5* gene accounts for approximately 80%–85% of Alport syndrome cases. Autosomal recessive inheritance caused by mutations in the *COL4A3* or *COL4A4* genes accounts for approximately 15% of Alport syndrome cases. Autosomal dominant inheritance of Alport syndrome is very rare with the pathogenic genes also being *COL4A3* or *COL4A4*.^65^

The main manifestation of Alport syndrome is the structural abnormality of the GBM caused by the functional deficiency of collagen type IV. Collagen type IV is composed of six different α‐chains that assemble into three different heterotrimers (α1α1α2, α3α4α5, and α5α5α6) that have a tissue‐specific distribution.^67^ These heterotrimers play a critical role in the structure and function of the basement membrane. The α3α4α5 heterotrimer is exclusively produced by podocytes in the GBM.^66^ Therefore, podocytes are considered a crucial cell type affected by Alport syndrome pathology. Patients with Alport syndrome commonly experience proteinuria due to the disruption of their filtration barrier. This is caused by altered podocyte orientation, effacement, and slit diaphragm breakdown. All of which are directly related to the critical role of the GBM as a component of the filtration barrier.

The course of Alport nephropathy includes numerous distinct stages, beginning with isolated hematuria and gradually progressing to moderate albuminuria, severe proteinuria, and ultimately resulting in a decline in glomerular filtration rate (GFR). The time interval between these stages can differ substantially among patients, primarily influenced by sex and *COL4A* genotype.

The current goal of therapy for Alport syndrome is to effectively extend the time between the progression stages as much as possible while maintaining patient safety. The use of angiotensin‐converting enzyme inhibitors in patients with Alport syndrome has been found to delay the onset of renal replacement therapy and increase life expectancy, especially when initiated at an early stage of the disease. This benefit is greater in patients with isolated hematuria or microalbuminuria.^68^ Additionally, limiting protein intake can ease the strain on the glomerular filtration membrane and slow disease progression. Symptomatic treatment can help alleviate symptoms, such as anemia, hypertension, and edema. Dialysis and kidney transplantation are the primary treatment options for end‐stage renal disease, both of which can replace kidney function and improve patients' quality of life.^67^

Gene therapy is an ideal indication for Alport syndrome, and there are currently preclinical and clinical studies underway (Table 1). Lademirsen is a drug that targets microRNA‐21. Preclinical studies and phase 2 clinical trials have demonstrated its ability to reduce miR‐21 levels and alleviate the progression of Alport syndrome.^69^ Yamamura et al. show that ASOs can induce exon skipping to alleviate the progression of Alport syndrome.^70^ Exon 21 skipping resulted in the production of truncated functional protein that facilitated collagen re‐deposition and alleviated proteinuria in mice with the *Col4α5* ^*R471X*^ mutation.^70^, ^77^ Daga et al. have reported using CRISPR/Cas9 for homologous recombination repair of mutation sites in patients' urinary podocytes in vitro, with a very high correction rate ranging from 44% in the *COL4A3* gene to 58% in the *COL4A5* gene, and a decrease in insertion‐deletion percentage (10.4% for *COL4A3* and 8.8% for *COL4A5*).^72^ Lin et al. have shown that specific induction of Col4α3 expression in podocytes of *Col4α3* ^*−/−*^ mice can lead to the re‐expression of the α3α4α5(IV) trimer and alleviate kidney dysfunction, prolonging the lifespan of mice.^71^ However, the specific induction of Col4α3 expression in the glomerular endothelial cells of *Col4α3* ^*−/−*^ mice cannot induce the re‐expression of the α3α4α5(IV) trimer.^78^ These studies propose that podocytes play a critical role in the re‐expression of the α3α4α5(IV) trimer. Therefore, future research on gene replacement therapy for Alport syndrome needs to specifically deliver therapeutic transgenes to podocytes. Heikkila et al. have demonstrated the successful delivery of the *COL4A5* gene into swine kidneys using an adenovirus vector, leading to successful deposition of COL4A5 in the GBM^73^ (Table 1). This indicates that adenovirus has the potential to deliver full‐length *COL4A5* to the GBM, but the strong immunogenicity of adenovirus still presents challenges for its clinical applications. Gene therapy aims to correct or replace the mutated genes responsible for Alport syndrome with the potential to halt or even reverse the progression of the disease.

**TABLE 1:** Gene therapies currently in development for pediatric genetic kidney diseases.

Disease	Treatment method	Target	Status	Intervention
Alport syndrome	Anti‐microRNA‐21 drug (Lademirsen)	microRNA‐21	Clinical trial	Weekly subcutaneous injection^69^
	Antisense oligonucleotide	COL4A5	Clinical trial	Subcutaneous treatment^70^
	Transgenic mouse	COL4A3	Preclinical study	Inducible^71^
	CRISPR/Cas9 mediated HDR	COL4A3/5	Preclinical study	AAV (in vitro)^72^
	Therapeutic transgenes	COL4A5	Preclinical study	Adenovirus (swine model)^73^
PKD	Transgenic mouse	PKD1/PKD2	Preclinical study	Inducible^74^
	Transgenic mouse	PC1‐CTT	Preclinical study	Inducible^75^
	Anti‐microRNA‐17 drug (RGLS4326)	microRNA‐17	Preclinical study	Subcutaneous injection^76^

### 3.2 Current therapies for PKD

PKD can be classified into two types based on inheritance: autosomal dominant polycystic kidney disease (ADPKD) and autosomal recessive polycystic kidney disease (ARPKD). While ADPKD is typically thought of as an adult‐onset disease, research has shown that it can be diagnosed in childhood or even in the fetal stage, and fetal‐onset cases tend to be more severe than adult‐onset cases.^79^, ^80^

#### 3.2.1 ADPKD

ADPKD is a common inherited kidney disease characterized by the formation of multiple fluid‐filled cysts in the kidneys. The global incidence of ADPKD is approximately 1 in 400 to 1000 people.^81^ ADPKD is caused by mutations in the *PKD1* or *PKD2* genes, which lead to a loss of function of the proteins they encode, polycystin‐1 and polycystin‐2, respectively.

The *PKD1* gene, which is responsible for approximately 78% of ADPKD cases, encodes for polycystin‐1, a large transmembrane protein expressed in the renal tubular epithelial cells. Polycystin‐1 forms a complex with polycystin‐2 on the extracellular side of the cell membrane, and together they participate in biological processes, such as cell signaling and apoptosis.^82^ Mutations in the *PKD1* gene can result in abnormal function of polycystin‐1, leading to abnormal cell proliferation, fluid secretion, and secretion pathway regulation, ultimately causing the occurrence and development of ADPKD.^82^

The *PKD2* gene, which is responsible for approximately 15% of ADPKD cases, encodes for polycystin‐2, an ion channel protein located on the endoplasmic reticulum and cell membrane of renal tubular cells. Polycystin‐2 forms a complex with polycystin‐1 and participates in biological processes such as regulation of calcium ion channels and cell signaling.^83^, ^84^ Mutations in the *PKD2* gene can result in the abnormal function of polycystin‐2, affecting the regulation of calcium ion concentration within cells and leading to abnormal cell proliferation and secretion pathway regulation, ultimately causing the occurrence and development of ADPKD.^84^

In addition to the kidneys, ADPKD can affect other organs, such as the liver, pancreas, and spleen. The severity of ADPKD can vary greatly between individuals with some people experiencing few or no symptoms and others developing ESKD and requiring dialysis or a kidney transplant.

Currently, there is no cure for ADPKD, but treatments can help manage symptoms and slow down the progression of the disease. Lakhia et al. found that miR‐17 inhibits the expression of PC1 or PC2 proteins in ADPKD cystic cells.^76^ Inhibiting miR‐17 with oligonucleotide RGLS4326 can substantially restore the expression levels of PC1/PC2, alleviating the disease progression of ADPKD.^76^ Dong et al. developed a mouse model that allowed them to turn off either *Pkd* gene in adult mice and then reactivate the gene at a later time. Their findings demonstrate that when the *Pkd* genes are re‐expressed in cystic kidneys, there is a quick reversal of ADPKD.^74^ Recently, Onuchicet al. demonstrated that the transgenic expression of the final 200 amino acids of PC1 in ADPKD mouse models suppressed cystic phenotype and preserved renal function^75^ (Table 1). This suggests that re‐expressing polycystin or polycystin truncated protein in ADPKD may be a potential therapeutic approach and is worth explored further.

#### 3.2.2 ARPKD

ARPKD is a severe genetic disorder that causes cyst formation in both kidneys and congenital hepatic fibrosis. It is estimated to affect approximately 1 in 20,000 to 40,000 live births. ARPKD usually presents during the perinatal or childhood period and is a major contributor to pediatric morbidity and mortality.^85^ Other common symptoms include hypertension, hepatic cysts, pancreatic cysts, urinary tract infections, renal failure, abdominal masses, etc. In infancy, ARPKD can also manifest as pulmonary hypoplasia, renal dysfunction, malnutrition, etc. The clinical presentation of ARPKD varies depending on the patient's age, disease severity, and presence of complications.

*PKHD1* is currently the only known disease‐causing gene for ARPKD and encodes a protein composed of 4074 amino acids, a single transmembrane receptor‐like protein called fibrocystin.^85^ It is found in cilia of epithelial cells lining the tubules of the kidneys and bile ducts in the liver. Fibrocystin plays a critical role in regulating cellular processes, such as cell division, differentiation, and signaling.^86^, ^87^

Currently, the main treatment approach for ARPKD focuses on managing the complications of the disease to alleviate symptoms and improve patients' quality of life. These complications include high blood pressure, liver complications, urinary tract infections, among others. Treatment options include medication, surgical intervention, and kidney transplantation.^88^, ^89^ However, the effectiveness of treatment methods varies depending on the patient's age, severity of the disease, and treatment selection. In some cases, ARPKD can lead to kidney failure, where kidney transplantation may be the only treatment option.

There is currently no gene therapy strategy for ARPKD in clinical research. Multiple factors have led to the stagnation of ARPKD gene therapy drug development. ARPKD progresses rapidly in patients and often occurs during the perinatal and neonatal periods. Currently available ARPKD disease models do not simulate clinical features well, which make it difficult to develop effective gene therapy strategies. Therefore, there is an urgent need for the development of gene therapy drugs for ARPKD.

## 4 PEDIATRIC GENETIC KIDNEY DISEASE GENE THERAPY: FUTURE DIRECTIONS AND CHALLENGES

### 4.1 Future directions

**FIGURE 2:**
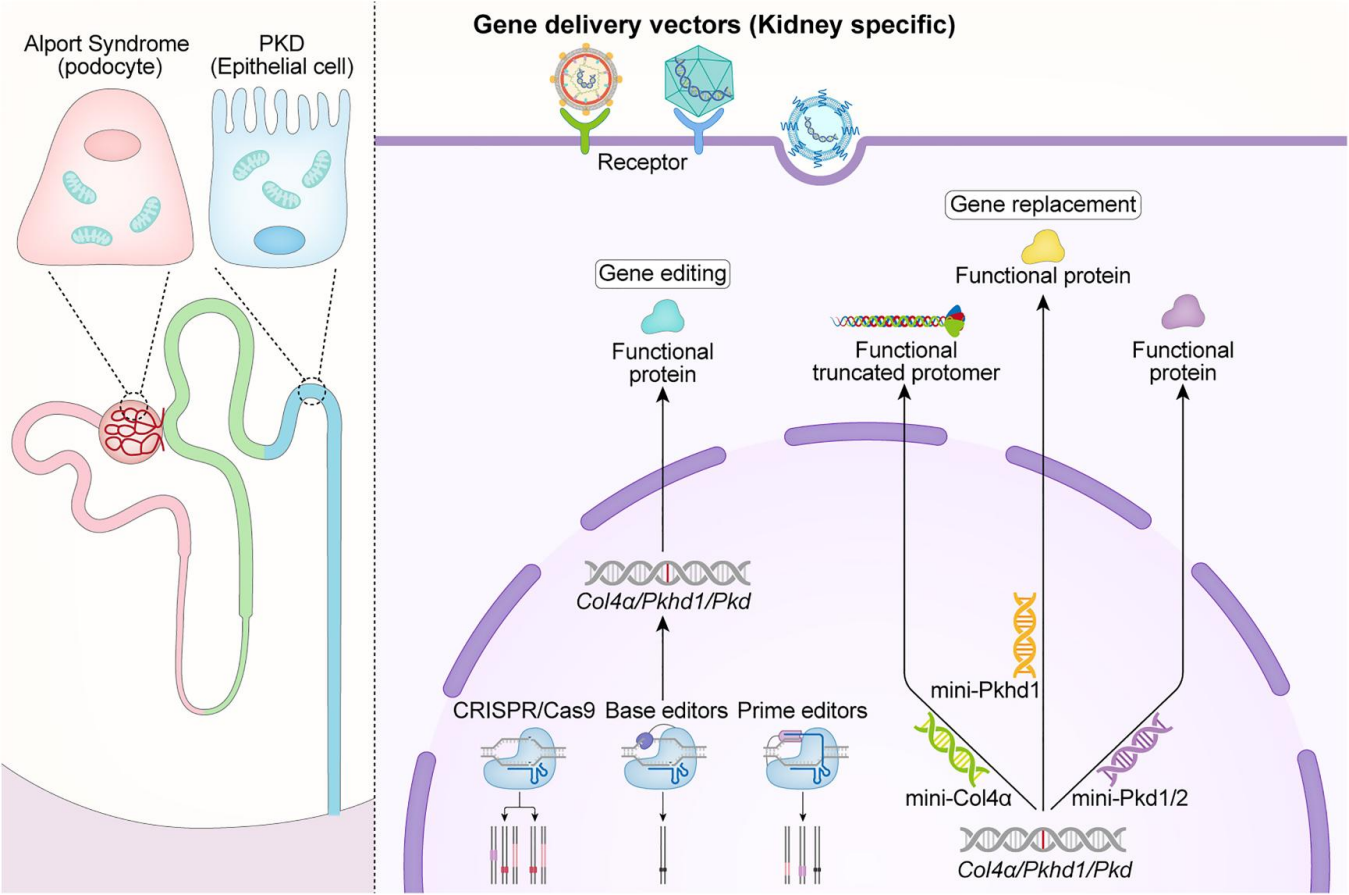
Future directions of gene therapy for pediatric genetic kidney diseases. Genetic kidney diseases in children affect specific cells with Alport syndrome predominantly affecting podocytes and PKD affecting tubular cells. Treatment options vary depending on the affected cells and mutation types. Base editors can repair single nucleotide mutations, while prime editing systems and CRISPR/Cas9‐mediated homologous recombination can fix fragment deletions and insertions. For patients with different mutation types, developing functional genes is a suitable approach. For Alport syndrome and PKD, gene therapy with truncated functional genes can be developed.

At present, the available treatments (non‐genetic) for Alport syndrome only provide temporary relief from the disease and do not restore sustained collagen expression. ASO therapy can generate partially functional protein, but it requires continuous administration and is limited by a high requirement for specific mutation sites. In theory, CRISPR/Cas9 and its related technologies, such as BEs and PEs, have the potential to correct various types of type IV collagen mutations, including point mutations, fragment deletions, insertions, and replacements (as illustrated in Figure 2). However, current research has only demonstrated the possibility of CRISPR/Cas9‐mediated homologous recombination repair of COL4A3/5 mutations in vitro.^72^ The efficiency of this method in vivo depends on the cell cycle phase, and for non‐proliferating cells such as podocytes, the efficiency is very low. Therefore, it remains unclear whether CRISPR/Cas9 can achieve effective homologous recombination repair in vivo. In contrast, gene repair mediated by BEs and PEs is not dependent on the cell cycle phase and has a higher efficiency in vivo than homologous recombination repair. However, their larger editing components result in low delivery efficiency in vivo. To address this issue, researchers are currently developing more efficient and smaller versions of BEs and PEs.

Currently, there are no gene editing strategies for PKD being investigated in clinical research. While CRISPR/Cas9‐based gene editing tools have the potential to repair *PKD/PKHD1* mutations, developing disease models that accurately reflect the clinical features of PKD is necessary. However, repairing *PKD/PKHD1* mutations differs markedly from repairing COL4A mutations. Repairing *COL4A* mutations leads to the production and secretion of functional collagen protein, which directly alleviates disease progression by increasing collagen deposition. In contrast, *PKD/PKHD1* mutations activate downstream events, such as cyclic adenosine monophosphate (cAMP), that promote cyst formation and cell proliferation,^82^, ^90^ making it unclear whether repairing *PKD/PKHD1* mutations can alleviate abnormal activation of downstream events. Abnormal activation of cAMP has been observed to impact aberrant transcriptional regulation in diverse PKD disease models.^90^, ^91^, ^92^ Further studies are required to determine whether repairing *PKD/PKHD1* mutations can alleviate this abnormal activation.

Effective correction of mutated genes in patients with Alport syndrome can be achieved through gene editing. However, designing suitable gene editing systems for different types of mutations can increase the cost and duration of drug development, and gene editing still faces potential off‐target problems. On the other hand, gene replacement therapy can be applied to all patients with Alport syndrome by delivering full‐length collagen type IV to treat type IV collagen deficiency. This therapy is promising, but maintaining the sustained expression of exogenous genes in target cells while avoiding genome integration is a challenge. AAV vectors are preferred for this therapy due to their low integration capability and long‐term expression ability, but their packaging capacity is limited to 4.9 kb, which makes it difficult to express collagen type IV proteins that are approximately 5.1 kb in length.^53^, ^54^ In the case of DMD, gene therapy using AAV to deliver functional exogenous genes is a promising strategy, but the dystrophin gene's coding sequence of 14 kb exceeds the packaging size limit of AAV. To overcome this limitation, researchers have developed truncated micro‐dystrophin proteins by deleting non‐essential structural domains from its protein sequence, which retain their function.^93^ Therefore, the development of functional mini‐collagen IV genes for gene replacement therapy also holds promise as a therapeutic strategy for Alport syndrome.

Collagen type IV's protein structure comprises three primary segments: the 7s domain, the collagen domain, and the NC1 domain. The NC1 domain plays a critical role in the assembly of collagen trimers, whereas the 7s domain is crucial for assembling different trimers in the basement membrane. The middle collagen region comprises a repetition of Gly‐X‐Y.^94^, ^95^, ^96^ Research results from ASO treatment indicate that eliminating a part of the collagen region has no impact on trimer formation and assembly.^70^ Thus, deleting the Gly‐X‐Y region may be a potential direction for creating functional truncated collagen proteins in the future (refer to Figure 2).

The length of the *PKD/PKHD1* genes exceeds the packaging capacity of AAV. To develop miniaturized version of the *PKD/PKHD1* genes that meets AAV packaging requirements, it is necessary to understand the functional domains of PC1/PC2/FPC. PC1 is a very large 462‐kDa membrane glycoprotein and is cleaved at both its N‐ and C‐termini.^97^ C‐terminal cleavage of PC1 generates several shorter PC1 CTF fragments and a C‐terminal tail fragment (PC1‐CTT).^98^, ^99^ Lin et al. have demonstrated that the expression of a protein construct containing a 17‐kDa PC1‐CTT fragment rescued the fragmented mitochondrial network observed in PC1‐deficient cells.^99^ These findings suggest that PC1‐CTT has the potential to modify mitochondrial function in vivo and could potentially alleviate the progression of PKD disease. FPC is a receptor‐like single‐pass transmembrane glycoprotein with a short cytoplasmic C‐terminal tail.^100^ FPC contains motifs associated with ciliary targeting, nuclear translocation, and interaction with PC2.^101^, ^102^ The functional validation of delivering the C‐terminus of FPC to mitigate ARPKD disease progression needs to be verified. The functions and mechanisms of *PKD/PKHD1* C‐terminus require further exploration. Confirming its function may facilitate the development of AAV‐mediated gene therapy drugs for PKD (Figure 2).

### 4.2 Current challenges

Gene therapy has emerged as a promising approach for treating genetic kidney diseases in children. However, the biggest bottleneck in developing gene therapy drugs for these diseases is achieving efficient delivery of the therapeutic genes to the kidneys.

Current gene therapy vectors, such as AAVs and LNPs, have limitations in their ability to target the kidneys effectively.^103^, ^104^ This is due in part to the glomerular filtration barrier, which retains many gene vectors, and the complex structure of the kidneys, which makes it difficult to target specific types of kidney cells affected by different genetic kidney diseases.

To address this challenge, researchers are exploring in situ delivery techniques for the kidneys, which involve delivering the therapeutic genes directly to the kidneys in their native environment.^105^ These techniques include renal artery injection, retrograde renal vein injection, subcapsular injection, and retrograde ureteral injection.

Renal artery injection is known to be highly efficient in reaching the glomerulus in pig models, but it is a challenging procedure to perform and can cause damage to the kidneys.^73^ Subcapsular injection is another method that involves injecting the gene vector under the kidney capsule, but its efficiency depends on the number and location of injections. Additionally, gene delivery vectors used in subcapsular injection are typically limited to the injection area, mainly in the renal cortex, making it unsuitable for effective gene therapy.

Retrograde ureteral injection is another approach, which involves injecting the gene vector through the ureter in a retrograde fashion. However, this method may cause irreversible damage to the ureter and potential obstruction, and it is believed to have limited delivery efficiency to proximal tubule cells and collecting duct cells.^106^

The ultimate goal is to develop safe and efficient gene delivery vectors that can target different types of kidney cells affected by different genetic kidney diseases. To achieve this goal, new targeted gene delivery vectors need to be developed that can overcome the challenges of delivering genes to the kidney and can be delivered through minimally invasive procedures with minimal side effects. Such vectors would allow for effective gene therapy and treatment of a range of genetic kidney diseases.

## 5 CONCLUDING REMARK

The management of genetic kidney diseases in children necessitates a comprehensive approach, and gene therapy represents a critical resource for addressing these ailments. The gene‐editing toolbox has greatly expanded with the advent of CRISPR/Cas9 technology, enabling the correction of any mutation type. Designing targeted delivery vectors for specific kidney cells to deliver gene editors and functional genes will be the primary challenge in developing gene therapy drugs for pediatric genetic kidney diseases in the future.

## AUTHOR CONTRIBUTIONS

Yi Lu wrote the manuscript; Yi Lu and Yandong Song prepared the figures; Shaokai Sun edited the manuscript; Lirong Zhang and Yupeng Chen conceived and wrote the manuscript.

## CONFLICT OF INTEREST STATEMENT

Yuping Chen is the member of the *Pediatric Discovery* Editorial Board, who was excluded from all editorial decision‐making related to the acceptance of this article for publication. The remaining authors declare no conflict of interest.

## ETHICS STATEMENT

Not applicable.

## Data Availability

Data sharing is not applicable to this article as no new data were created or analyzed in this study.
